# Conserved DNA sequence features underlie pervasive RNA polymerase pausing

**DOI:** 10.1093/nar/gkab208

**Published:** 2021-03-31

**Authors:** Martyna Gajos, Olga Jasnovidova, Alena van Bömmel, Susanne Freier, Martin Vingron, Andreas Mayer

**Affiliations:** Otto-Warburg-Laboratory, Max Planck Institute for Molecular Genetics, Berlin 14195, Germany; Department of Mathematics and Computer Science, Freie Universität Berlin, Berlin 14195, Germany; Otto-Warburg-Laboratory, Max Planck Institute for Molecular Genetics, Berlin 14195, Germany; Department of Mathematics and Computer Science, Freie Universität Berlin, Berlin 14195, Germany; Department of Computational Molecular Biology, Max Planck Institute for Molecular Genetics, Berlin 14195, Germany; Otto-Warburg-Laboratory, Max Planck Institute for Molecular Genetics, Berlin 14195, Germany; Department of Computational Molecular Biology, Max Planck Institute for Molecular Genetics, Berlin 14195, Germany; Otto-Warburg-Laboratory, Max Planck Institute for Molecular Genetics, Berlin 14195, Germany

## Abstract

Pausing of transcribing RNA polymerase is regulated and creates opportunities to control gene expression. Research in metazoans has so far mainly focused on RNA polymerase II (Pol II) promoter-proximal pausing leaving the pervasive nature of pausing and its regulatory potential in mammalian cells unclear. Here, we developed a pause detecting algorithm (PDA) for nucleotide-resolution occupancy data and a new native elongating transcript sequencing approach, termed nested NET-seq, that strongly reduces artifactual peaks commonly misinterpreted as pausing sites. Leveraging PDA and nested NET-seq reveal widespread genome-wide Pol II pausing at single-nucleotide resolution in human cells. Notably, the majority of Pol II pauses occur outside of promoter-proximal gene regions primarily along the gene-body of transcribed genes. Sequence analysis combined with machine learning modeling reveals DNA sequence properties underlying widespread transcriptional pausing including a new pause motif. Interestingly, key sequence determinants of RNA polymerase pausing are conserved between human cells and bacteria. These studies indicate pervasive sequence-induced transcriptional pausing in human cells and the knowledge of exact pause locations implies potential functional roles in gene expression.

## INTRODUCTION

RNA polymerase II (Pol II) is the key enzyme responsible for the transcription of all nuclear protein-coding and a large set of non-coding genes in eukaryotic cells (1–3). Pol II transcription is not only restricted to genes but also widespread in intergenic regions (3–5). Transcription is generally subdivided into three distinct phases: initiation, elongation and termination (1,6). Following the recruitment of Pol II to the gene promoter, the DNA is unwound to form a ‘transcription bubble’ allowing the single strand DNA template to bind the active site and to enable transcription initiation. When the RNA-DNA hybrid in the active center cleft reaches a length of 8 nt, it is stably bound (7) and Pol II can enter into the elongation phase. During elongation, Pol II translocates along the DNA template in single nucleotide steps while the nascent RNA chain grows accordingly by one nucleotide at a time (8,9). The incoming nucleoside triphosphate (NTP) binds to the free insertion site (+1 position). Following the formation of a phosphodiester bond, Pol II moves to the next DNA template position and a new nucleotide-addition cycle can start (8,9).

The established view that transcription initiation was the major regulatory step in the synthesis of nascent RNA was held for decades (10). This view was challenged by the discovery of widespread transcriptional pausing in the promoter-proximal region, 50–150 nt downstream of the transcription start site (TSS), of a large set of genes in flies and mammalian cells (11–16). Pauses interrupt the nucleotide-addition cycle and evidence has accumulated that promoter-proximal pausing is strictly regulated indicating the importance of post-initiation transcription regulation in metazoan gene expression (17,18). Research by many groups has provided insights into the molecular mechanisms of pausing regulation. These studies revealed over a dozen of *trans*-acting factors that are involved in the establishment and release of promoter-proximal pausing by Pol II in mammalian cells (17,18).

Besides of *trans*-acting factors, *cis*-DNA motifs, although less studied, have been implicated in transcriptional pausing. DNA sequence-induced pausing is best characterized in bacteria (19,20). In bacteria, a consensus DNA sequence was uncovered that underlies ubiquitous transcriptional pausing (21–23). DNA motifs have recently also been linked to Pol II promoter-proximal pausing in metazoans. A consensus DNA motif was identified in early Drosophila embryos that underlies a large set of promoter-proximal pauses, termed the ‘pause button’ (24). Recently, two studies in human cells identified a GC-rich sequence motif involved in promoter-proximal pausing (25,26). Despite these observations, the sequence determinants of transcriptional pausing in mammalian cells still remain obscure. Moreover, it is unclear whether the DNA sequence features of pausing are conserved in evolution.

Pol II can also halt during elongation outside of promoter-proximal gene regions. Since the vast majority of studies have exclusively focused on the regulation of promoter-proximal pauses, only little is known about Pol II pauses at other genomic locations (27). Evidence is accumulating that Pol II often pauses at intron-exon boundaries in mammalian cells, flies and yeast suggesting a potential role in coordinating transcription with co-transcriptional splicing (11,12,28–30). Moreover, Pol II pausing was observed at the 3′-end of a large set of mammalian protein-coding genes that implies a potential function in 3′-RNA processing and transcription termination (31–34). Recently, Pol II pausing was also detected at transcribed enhancer regions (35). Despite these findings, the widespread nature of Pol II pausing outside of the promoter-proximal gene regions is unknown.

New genome-wide approaches that provide a quantitative measure of transcriptionally engaged RNA polymerase at nucleotide resolution now enable the analysis of the full spectrum of transcriptional pausing in cells. One approach, called native elongating transcript sequencing (NET-seq), relies on deep sequencing of the 3′-ends of the nascent RNA providing DNA strand-specific Pol II densities with single-nucleotide resolution (11,28,36). Although NET-seq was originally developed for yeast (36), protocols became available for other species including mammalian cells (11,28), plants (37,38) and bacteria (21–23). NET-seq is especially powerful for studying pausing because it captures all states of transcribing RNA polymerase: paused, recovering from pause and transcribing (11). Potential pause sites emerge as genomic nucleotide positions at which NET-seq signals peak and where Pol II is captured with a higher probability (27). Despite the availability of these genomic approaches for profiling Pol II densities at single-nucleotide resolution, a systematic overview of pausing sites is still missing. This is mainly due to the lack of computational tools that permit robust peak detection from nucleotide-resolution occupancy data and because of inherent limitations of common occupancy profiling approaches leading to artifactual peaks (39–41) misinterpreted as pausing sites.

In this combined computational and experimental study, we investigated the pervasive nature of transcriptional pausing primarily in human cells and the role of DNA sequences as potential pause determinants. We developed a new algorithm to identify peaks with high precision from nucleotide resolution occupancy profiling data, complemented by the development of a NET-seq approach that minimizes the formation of artifactual signal peaks commonly misinterpreted as pausing locations. Application of this peak caller on NET-seq data provides an atlas of transcriptional pausing sites with single-nucleotide resolution in human cells. Notably, this analysis uncovers pervasive transcriptional pausing of Pol II outside of promoter-proximal gene regions and a new consensus pause motif underlying promoter-proximal pauses. Machine learning modeling reveals the DNA sequence features that are predictive for pausing. Interestingly, key determinants for DNA sequence-induced transcriptional pauses are conserved from bacteria to human cells.

## MATERIALS AND METHODS

### Cell lines and cell culture conditions

HEK293T (ATCC, cat. nr. CRL-11268) and HeLa S3 (ATCC, cat. nr. CCL-2.2) cells were cultured in DMEM (ThermoFischer Scientific) containing 10% FBS Superior (Biochrom) and 5% penicillin–streptomycin (ThermoFischer Scientific).

### Cell fractionation and library preparation for NET-seq and nested NET-seq

Cell fractionation and nascent RNA extraction were performed as described previously (42). The library preparation for standard NET-seq was conducted as described previously (42) with the following modifications. The ‘barcode DNA oligo’ was modified to now include a random decamer sequence instead of a random hexamer (5′-rApp/(N)_10_CTGTAGGCACCATCAAT/3′-ddC; 5′-rApp: 5′-riboadenylate; 3′-ddC, 3′-dideoxycytidine). Due to this increase in the length of the oligo, the size selection of ligated RNA, cDNA and the PCR product was adapted to 60–130 nt, 100–180 nt and 150–200 nt, respectively. For the nested NET-seq library preparation, the following additional modifications were introduced. For library preparation, 1 μg RNA was used as an input per sample. Reverse transcription was performed as described before with the following primer: 5′-Phos/ATCTCGTATGCCGTCTTCTGCTTG/iSp18/CACTCA/iSp18/TCCGACGATCATTGATGGTGCCTA-3′ (iSp18: internal 18-atom hexa ethylenglycol spacer (42). The specific depletion of highly abundant mature RNAs was omitted. After the circularization, the ssDNA was purified using the ZYMO Clean & Concentrator-5 Kit according to the manufacturer’s instructions. The final PCR amplification of the nested NET-seq library was conducted using the Phusion high-fidelity (HF) DNA polymerase (NEB), and the following primers: forward index primer: 5′-AATGATACGGCGACCACCGAGATCTACACGATCGGAAGAGCACACGTCTGAACTCCAGTCAC-index-of-choice-TCCGACGATCATTGATGGTGCCTA*C*A*G-3′; reverse primer oNTI231: 5′-CAAGCAGAAGACGGCATACGA-3′. The forward index primer contained an 8 nt Illumina index of choice and phosphorothioate bonds between the 3′-most four bases (indicated by an asterisk) to prevent cleavage by the 3′ to 5′ exonuclease activity of the Phusion HF DNA polymerase. The nested libraries were amplified by 6 to 8 PCR cycles. The PCR product was purified from a 4% E-Gel™ EX Agarose-Gel (Thermofisher Scientific) using the NucleoSpin Gel & PCR clean-up (Macherey-Nagel) according to the manufacturer’s instructions. Libraries were sequenced in SR100 mode on an Illumina NovaSeq 6000 sequencing system using the custom primer oLSC006 (5′-TCCGACGATCATTGATGGTGCCTACAG-3′) (42).

### NET-seq and nested NET-seq data processing

Data processing of NET-seq was performed as described in (11), with the following modifications. To remove PCR duplicates, identical reads that also contain the same unique molecular identifier (UMI) were collapsed to one read using the DNA sequence clustering software Starcode (43). The ten 5′-end nucleotides corresponding to the UMI were trimmed, but the information of the barcode remained associated with the read using a custom Python script. For all samples, reads were aligned to the human reference genome (GRCh38.p12) using the STAR aligner (v2.4.0) (44) with the following parameters: –clip3pAdapterSeq ATCTCGTATGCCGTCTTCTGCTTG –clip3pAdapterMMp 0.21 –clip3pAfterAdapterNbases 1 –outFilterMultimapNmax 1 –outSJfilterOverhangMin 3 1 1 1 –outSJfilterDistToOtherSJmin 0 0 0 0 –alignIntronMin 11 –alignEndsType EndToEnd. For uniquely mapped reads, the position corresponding to the 3′-end of the nascent RNA fragment (the 5′-end of the sequencing read after removal of the barcode) was recorded with a custom Python script using the HTSeq package (45). As NET-seq signals at the 3′-most nucleotide position of introns and exons can be due to pausing of Pol II at this site or because of splicing intermediates (11), reads that aligned with their 3′-ends precisely to these nucleotide positions (including the pA site) were discarded prior to subsequent analyses. We masked regions of the human reference genome that are transcribed by RNA polymerase I and III as well as the following short chromatin-associated RNA species: 5S, 7SK, HY1, HY3, HY4, HY5, LSU-rRNA_Hsa, RNase_MRP_RNA, RNase_P_RNA, SSU-rRNA_Hsa, U1, U2, U3, U4, U5, U6, U7, U8, U13, U14, U17, Y_RNA, antisense_RNA, guide_RNA, miRNA, misc_RNA, rRNA, rRNA_pseudogene, sRNA, scRNA, snRNA, snoRNA, tRNA, telomerase_RNA and vaultRNA. The regions were determined using the following annotations: GENCODE (v28 and v29), RefSeq release 109, miRBase v22.1 and the UCSC’s RepeatMasker.

### *In silico* identification and removal of reverse transcription artifacts

For standard NET-seq, reverse transcription mispriming events were identified and removed when the UMI sequence matched exactly the genomic sequence downstream of the aligned read. Additionally, the signal at these nucleotide positions was masked if >5% of reads that aligned to these sites originated from reverse transcription mispriming. Here, we discovered a substantial fraction of reads with alterations in the UMI sequence that escaped detection before. These changes most likely arose from errors introduced during reverse transcription, PCR or during Illumina sequencing. These reads were discarded prior to further analyses.

#### Quantification of mispriming events

We defined mispriming prone positions as nucleotide positions where >5% of the aligned reads originated from RT mispriming. Next, the number of mispriming events was estimated by the number of reads (PCR duplicates excluded) that aligned to mispriming prone positions and for which the UMI sequence had at least a 50% similarity with the genomic sequence downstream of the aligned read. The percentage of RT mispriming events that escaped detection was calculated as the ratio of mispriming events for which the UMIs were not identical with the genomic sequence and all mispriming events (including reads with UMIs exactly matching the genomic sequence).

### Pause detecting algorithm (PDA)

The algorithm was designed for detecting signal intensity peaks from single-nucleotide resolution Pol II occupancy data corresponding to potential transcriptional pausing sites. The algorithm consists of two main steps: peak detection and peak evaluation. In the first step, all local maxima are detected. A local maximum is a nucleotide position *i* for which *x_i__−_*_1_< *x_i_* > *x_i_*_+1_, where *x_i_* is the signal intensity *x* at the position *i*. In the second step, a nonparametric resampling approach is applied for every detected peak to test if this peak has a significantly higher value than the expected value of the maximum given the local Pol II density. Local Pol II density for the position *i* is described by the number of reads *M* and the number of positions *l* with non-zero signal within the window of a given length *L* (here 200 nucleotides) centered at the position *i*. A value of local maximum is simulated by redistributing *M* reads over *l* positions and extracting the maximum number of reads per position from the newly obtained read distribution. The redistribution is conducted following the null hypothesis that Pol II does not accumulate at any position and a read has an equal probability of being assigned to each of the positions in the local window of length *l*. Such resampling generates a pool of *N* (here 10 000) simulated values of local maxima. Next, the *P*-value is estimated using the fraction of simulation experiments in which the simulated value of local maximum is higher than the observed local maximum. To control for multiple testing, all *P*-values are corrected using the Benjamini–Hochberg procedure. For further analyses, we considered pausing sites with a corrected *P*-value <0.05 as significant.

#### Reproducibility of pause detection by PDA

For calculating the percentage of significant peaks called at the same nucleotide position in NET-seq replicate measurements, only peaks for which a local maxima was detectable in all replicates were included. The percentage of significant peaks was calculated as the ratio of the number of significant peaks common in all replicates and the number of all significant peaks called in a given replicate only. For the two-sided Fisher exact test, all peaks were included regardless whether they were detectable in all replicates.

For comparison of pausing sites between different human cell lines, the reported percentage of signal intensity peaks that occur at the same nucleotide position was calculated as the ratio of peaks detected in both cell lines and the number of all peaks detected in the cell line with the lower sequencing depth.

### Assigning genomic regions to pausing sites

Based on their location, pausing sites were classified into one of four major categories: promoter-proximal, gene-body, antisense or intergenic. For defining the regions, we used GENCODE annotations (v28 and v29). A pausing site was classified as ‘promoter-proximal’ if located within 300 nucleotides downstream of the transcription start site (TSS). Pauses between +301 and the 3′-most polyadenylation (pA) site of a gene were classified as ‘gene-body’ pauses. If the pausing site was situated on the opposite strand of a gene in a region between 1000 nucleotides upstream of the most upstream TSS and the most downstream pA site, the pausing site was classified as ‘antisense’. In case of an overlap between the gene-body region of one gene and the antisense region of another gene, the pausing location remains undetermined. All pausing sites located outside the listed regions (promoter-proximal, gene-body, antisense) were classified as intergenic pauses.

The four main categories were further specified into their respective subclasses. Gene-body pausing sites were ‘exonic’ if they overlap with annotated exons, otherwise they were labeled ‘intronic’. Antisense pausing sites were categorized in ‘divergent’ if the pausing site was located upstream of the TSS or ‘convergent’ if it was downstream of the TSS overlapping with the gene-body. In case the pausing locations overlapped, the type of antisense pausing remained undetermined. Intergenic pausing sites occurred at the termination zone, located within 3.5 kb downstream of the pA site or at enhancer regions. The enhancer locations were determined using FANTOM5 enhancer annotation (46).

### Creating sets of pausing and non-pausing sites

We defined high-confidence pausing sites as pausing sites that were detected in all biological replicates at the same nucleotide position. Only high-confidence pausing sites were used for downstream analysis and to create a set of pausing sites (positive set). A set of non-pausing sites (negative set) was generated by sampling random positions at which pausing does not occur. To avoid creating artificial differences between both sets, a non-pausing site was sampled from the region [*x*, *x*+20] nucleotides downstream or upstream of a pausing location, where distance *x* depends on the region of the pausing site (50 for promoter-proximal pauses, 300 for pauses in other regions). Pausing and non-pausing sites are subsequently referred to as ‘genomic sites’.

### Genome-wide statistics

#### Annotation of active genes

For gene expression quantification, we used RSEM v1.3.1 (47) in the paired-end mode with the parameters ‘–star –calc-pme’, which uses the STAR v2.5.3a mapper. We considered a gene as actively transcribed when the observed steady-state RNA expression TPM (transcripts per million) was >1. ENCODE RNAseq data sets from HeLa (ENCFF000FQK, ENCFF000FQX, ENCFF000FQV, ENCFF000FQW, ENCFF000FOK, ENCFF000FOY, ENCFF000FOM, ENCFF000FOV) (48) were analyzed using the human assembly GRCh38.p12 and the annotated transcriptome GENCODE v28.

#### Comparison of pausing intensities

For the comparative analysis of signal intensities between promoter-proximal and gene-body pausing, only genes with pauses in both regions were included. Non-expressed genes were not explicitly excluded. However, the lowest TPM that was observed for example genes was >3.

#### Pausing in splice site proximity

Pausing sites were classified as ‘proximal’ when located within 40 nucleotides of a splice site, otherwise they were labeled ‘distant’. The proximal sites are further categorized into: ‘first exon-intron’, ‘intron-exon’, ‘exon-intron’ and ‘last intron-exon’ boundaries. If a pausing site was found in proximity of multiple boundaries, it was assigned to all categories.

#### Average pausing distance

For every gene with ≥2 pausing sites, we defined the average pausing distance as the mean distance between pausing sites that are located within the most upstream active TSS and the most downstream active pA site.

#### Gene ontology (GO) enrichment analysis

The GO enrichment analysis was conducted for genes with a short average pausing distance using over-representation analysis of the ConsensusPathDB package (49). Biological processes of genes with an average pausing distance of ≤1 kb were extracted using all active genes as a background and with a *P*-value cut-off of 0.05.

#### NET-CAGE analysis

NET-CAGE data available for HeLa cells were obtained from (50) using GEO accession GSM3318225. The conversion of genome coordinates between hg19 and hg38 human genome assemblies was done using CrossMap (51). The NET-CAGE signal density was calculated for three categories: promoter-proximal pausing sites, gene-body pausing sites and gene-body non-pausing sites. For each site, the signal density was calculated as the mean NET-CAGE signal per position within 200 bp upstream of a pausing or non-pausing site.

#### RNA 3′ end-seq analysis

RNA 3′ end-seq of nascent and newly synthesized (4sU-enriched), polyA(+) and polyA(-) RNA data available for HeLa cells were obtained for exosome depleted cells from (52) using GEO accessions: GSM4083152, GSM4083153. The obtained tracks were already pre-normalized between conditions using the signal at the last exon of highly expressed protein-coding genes to enable comparisons between conditions (52). The RNA 3′ end-seq signal density was calculated for four categories: promoter-proximal pausing, gene-body pausing, gene-body non-pausing and polyadenylation sites. The polyadenylation sites were derived from the same genes as for the pausing sites. For each site, the signal density was calculated as the mean RNA 3′ end-seq signal per position within a 20 bp window around a pausing or non-pausing site. The signals were averaged over biological replicates for each site. We estimated the amount of premature RNA cleavage and termination as the signal density obtained for RNA exosome (RRP40) depleted HeLa cells (52). A site was reported as a potential premature termination site, if the signal density at the location was higher than the 25th percentile of signal density at polyadenylation sites.

### Pausing sequence signatures

DNA sequences centered on genomic pausing sites were extracted. The corresponding non-pausing set was used to derive the background nucleotide distribution. Extracted pausing sequences and background frequencies were used to create enrichment logos with Logolas (53).

### Feature construction

Sequence characteristics that were previously implicated in pausing were included in the model. The determined features for all species included differences in nucleotide skewness and identity at positions of interest, thermodynamic features of the RNA-DNA hybrid, and free energy of the nascent RNA. A comprehensive list of features is given in Supplementary Table S1.

*XY* skewness is defined as $\frac{{| X |\ - \ | Y |}}{{| X |\ + \ | Y |}}$, where *|X|* denotes the number of nucleotides *X*. *X* and *Y* represent standard DNA nucleotides. A difference of XY skewness for all pairs of DNA nucleotides was calculated between the positions located 10 nucleotides upstream and downstream of the genomic site in a 20 nucleotide window.

The nucleotide identity was extracted from the +1, -1, -2, -3, -10, -11 position from the genomic site, corresponding to the active center of the RNA polymerase and both ends of the RNA-DNA hybrid.

Thermodynamic features, such as the entropy, enthalpy, Gibbs free energy and the melting temperature of the 10 nucleotide RNA-DNA hybrid, were calculated using MELTING (54) with the RNA-DNA model parameters.

The minimum free energy of the stretch of a nascent RNA between positions -11 and -29, where position -1 corresponds to the last nucleotide added to the nascent RNA, was calculated using the ViennaRNA package (55). The region between nucleotide -11 and -29 of the nascent RNA corresponds to the region of RNA hairpin structure formation (19).

Additional features were calculated for human cell lines since the following tools and databases were only available for human samples. The human-specific features include the DNA shape and form, binding motifs of transcription factors (TFs) and RNA-binding proteins (RBPs), and DNA methylation.

DNAshapeR (56) was used to calculate DNA shape descriptors including the minor groove width (MGW), roll, propeller twist (ProT), helix twist (HelT) and potential energy (EP). The features consist of minimum, maximum, mean, span and mean value of the first derivative of the descriptors calculated for the region 10 nucleotides upstream and 5 nucleotides downstream of the genomic site. The region encompasses the RNA-DNA hybrid and a 5-nucleotide long DNA fragment downstream of the polymerase active center.

The presence of non-B DNA forms in the region 100 nucleotides upstream or downstream of the genomic site was also taken into consideration. Regions of the human genome that tend to form non-B DNA structures such as Z-DNA, A-phased repeats, inverted repeats, mirror repeats and direct repeats were extracted from non-B database v2.0 (57). Additionally, pqsfinder (58) was used to predict the locations of G-quadruplexes.

The presence of a transcription factor binding motif (TFBM) was determined for three regions referred to as ‘polymerase footprint’ (25 nucleotides upstream and downstream of the genomic site), ‘upstream’ (100 nucleotides upstream of the polymerase footprint) and ‘downstream’ (100 nucleotides downstream of the polymerase footprint). About 810 position weight matrices (PWMs) describing 639 human transcription factors were downloaded from the JASPAR database (59). To limit the number of features added to the model, the PWMs were clustered into 111 consensus matrices using RSAT matrix-clustering (60). The consensus matrices were later used to scan for motif occurrences using the MOODS package (61).

About 1194 PWMs describing human RNA-binding protein (RBP) motifs were downloaded from ATtRACT database (62). To limit the number of features added to the model, the PWMs were clustered into 240 consensus matrices using RSAT matrix-clustering (60). The upstream region of a pausing site was scanned for motifs using the MOODS package (61). The RBP motif search was restricted to that region, because the complementary sequence corresponds to the nascent RNA that RBPs can bind.

In addition to purely sequence-dependent features, the DNA methylation levels were added to the model, as DNA methylation has previously been implicated in promoter-proximal pausing (63). Whole-genome shotgun bisulfite sequencing ENCODE data (ENCFF162HBC) obtained for HeLa cells was used for calculating the mean DNA methylation levels in the region encompassing 100 nucleotides upstream and downstream of the genomic site.

### Random forest classification model

Machine learning models were developed to distinguish pausing from non-pausing sites based on the genomic features. Each model was tuned, trained and tested on *n* sites, with two equally sized sets of pausing and non-pausing sites. The random forest classification model was implemented with the scikit-learn Python package and uses *p* predictor variables (genomic features), which showed a variable degree of correlation between each other. About 20% of the *n* sites were used to optimize the number of decision trees, the maximum number of levels, the minimum number of samples required to split a node and the minimum number of samples required at each leaf node. The model was trained and tested on the remaining 80% of observations using 10-fold cross-validation (5-fold for *Arabidopsis thaliana*). The model performance was assessed using the area under the curve (AUC) values of the precision-recall curve. The importance of each feature was computed as permutation importance. Variables with high positive values corresponded to important features for classification, whereas variables with values close to zero or negative corresponded to noise and were not informative. The number of sites *n*, the number of predictor variables *p* and the number of folds *k* together with additional parameters of each model are listed in Supplementary Table S2.

## RESULTS

### A robust peak caller for nucleotide-resolution occupancy profiling

**Figure 1.**
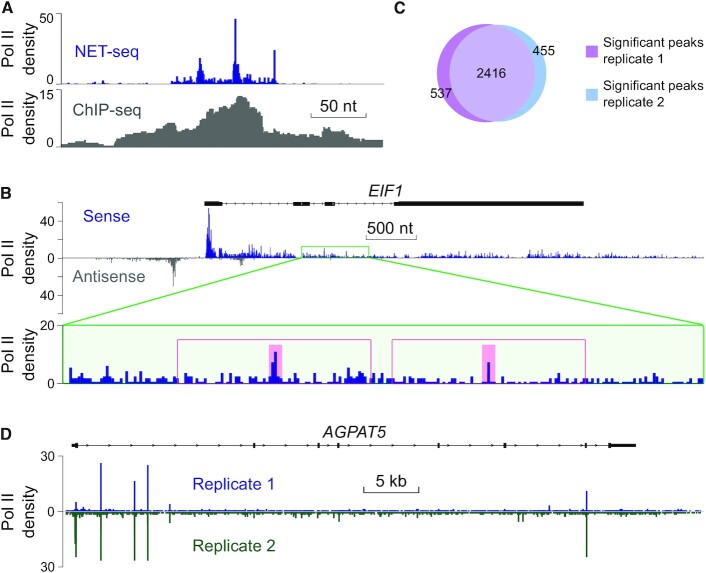
Development of a robust peak caller for single-nucleotide resolution genomic Pol II occupancy data. (**A**) Comparison of signal intensity peaks obtained by NET-seq and ChIP-seq. ChIP-seq data were generated by ENCODE (identifier: ENCFF144IVU) (48). The Pol II density is shown for a subregion (chrX: 71 555 028–71 555 500) of *OGT* obtained for HeLa cells. (**B**) Gene track view of peak identification using PDA. The top gene track shows NET-seq Pol II occupancy in both sense and antisense direction. Zoom-in view of a distinct genomic region (green rectangle) shows two local maxima (pink background) as revealed by PDA. Statistical importance is determined by comparing the Pol II occupancy at these positions with the expected local Pol II density value of the surrounding genomic region (red rectangle). (**C**) Venn diagram showing the overlap of significant peaks detected for technical NET-seq replicates obtained for human HEK293T cells. (**D**) Single gene example showing NET-seq signal intensity peaks occurring at the same nucleotide positions in biological replicate measurements obtained for HeLa cells.

Current peak calling algorithms that have been mainly developed for ChIP-seq data were not applicable to our NET-seq data sets because of the following reasons. First, the width and overall shape of Pol II occupancy peaks obtained from NET-seq and ChIP-seq strongly differed (Figure 1A), due to the much higher spatial resolution of NET-seq data. The high resolution allowed closely spaced peaks to be resolved that could not be deconvoluted by ChIP-seq (Figure 1A). Second, since ChIP-seq usually suffers from strong background signals, corresponding peak callers require control data sets as obtained by experiments using non-specific antibodies (64). Third, ChIP-seq peak callers rely on paired-end sequencing data (65), whereas NET-seq generates single-end sequencing data. To overcome these main limitations and to create an atlas of genomic transcriptional pausing sites with nucleotide resolution, we first developed a new algorithm, termed ‘pause detecting algorithm (PDA)’. Pause site detection using PDA is achieved in two subsequent steps. First, genomic regions with increased numbers of NET-seq reads are identified. PDA accomplishes this by identifying local maxima (Figure 1B). Second, a statistical model is used to assess the significance of called peaks (methods). Here, the significance of NET-seq read enrichment relative to the null hypothesis that reads are uniformly distributed within a transcribed region is determined.

To assess the robustness of peak calling from high-resolution occupancy data by PDA, we first applied our algorithm to technical NET-seq replicate data sets that had an almost identical sequencing coverage. These data allowed us to determine the reliability of peak calling by PDA irrespective of the sequencing depth and the biological variation. These data also allowed us to assess the potential impact of technical variation in NET-seq data on peak calling. Notably, 82% or 84% of significant peaks (*P*-value <2.2^−308^; Fisher exact test) of replicate 1 or 2 were called at the same nucleotide position, respectively (Figure 1C). This finding indicates that PDA calls high-resolution peaks reliably and that the effect of technical variation was minimal. We next applied PDA to biological NET-seq replicates (11). Interestingly, we found that 91% or 63% of significant peaks (*P*-value <2.2^−308^; Fisher exact test) occurred at the same nucleotide position in both replicates (Figure 1D and Supplementary Figure S1A). We extended our comparative analysis to NET-seq data available for other human cell lines including HEK293T (11) and MOLT4 (66). Interestingly, the majority of peaks (>55%) occurred at the same nucleotide position in different human cell lines (Supplementary Figure S1B). Under the assumption that NET-seq peaks are indicative for potential pausing sites as further detailed in the following section, these results suggest that Pol II pauses predominantly at defined nucleotide positions along genes and in the majority of cases at the same nucleotide in different cell types.

To learn about the sensitivity of peak calling using PDA, we randomly downsampled a high-coverage NET-seq data set available for HeLa cells (11) to simulate lower sequencing depths of NET-seq libraries. As expected, the number of called peaks correlated with the sequencing depth of NET-seq data and dropped proportionally to the decreasing number of sequencing reads (Supplementary Figure S1C). Since lowering the sequencing coverage unavoidably also reduces the library complexity, defined by the number of unique DNA fragments present in a given library, we cannot rule out that the drop in peak identification was partially caused by the decrease in library complexity. Together, these data show that PDA reliably calls high-confidence peaks from nucleotide-resolution genomic Pol II occupancy data.

### Nested NET-seq peaks inform on Pol II pausing sites at nucleotide resolution

**Figure 2.**
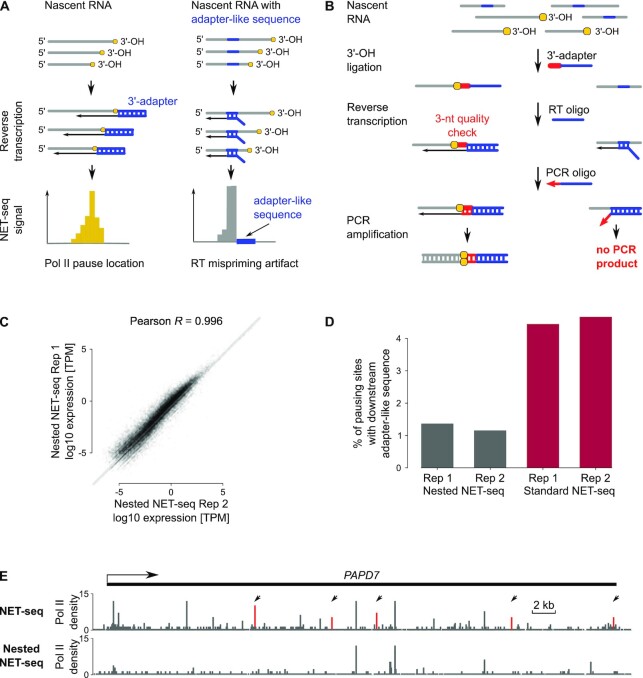
Eliminating artifacts originating from mispriming during reverse transcription (RT) by the development of the nested NET-seq approach. (**A**) Schemes for specific annealing of RT primer to ligated 3′-adapter (left) and non-specific annealing of RT primer to internal adapter-like sequences (right) during the RT reaction. The latter results in artifactual spikes of signal intensity. (**B**) Schematic view of the nested NET-seq library preparation illustrating how the PCR amplification of RT mispriming products is prevented. Briefly, the cDNA is synthesized using a nested RT primer (blue) that lacks the first three nucleotides (red) of the 3′-adapter creating a 3-nt quality control point. The amplification of RT-mispriming products is avoided by use of a reverse PCR primer that matches entirely to the 3′-adapter sequence. A 3-nt mismatch in case of RT-mispriming products prevents primer extension and amplification. (**C**) Correlation analysis of biological nested NET-seq replicates. The Pearson correlation coefficient (*R*) as indicated was derived from the Pol II occupancy of genes. (**D**) Number of potential artifactual signal intensity peaks due to RT-mispriming for standard and nested NET-seq data. Results for two biological replicate measurements are shown. (**E**) Gene tracks of standard NET-seq (top lane) and nested NET-seq (bottom lane) for a representative gene. Black arrows point to peaks (red) with downstream adapter-like sequences indicative for potential RT-artifacts.

It is generally assumed that peaks of signal intensities in Pol II profiling data correspond to high levels of genomic Pol II occupancy in cells. However, there is evidence that artifacts that originate from different steps during sequencing library preparation of genome-wide profiling methods can also result in peaks of signal intensity commonly misinterpreted as high occupancy levels (11,39–41). We next investigated which of the identified NET-seq peaks indeed reflected high levels of Pol II occupancy indicative for potential pausing sites. Previously, we found that a pileup of NET-seq reads can also descend from RNA processing intermediates, PCR duplicates and products of mispriming during reverse transcription (RT) (11). PCR duplication and RT mispriming (Figure 2A) represent common sources for artifacts in widely used genome- and transcriptome-wide profiling methods, including ChIP-seq and precision nuclear run-on and sequencing (PRO-seq), and represent no peculiarities of the NET-seq approach (39–41,67,68).

RNA processing intermediates, mainly due to splicing and 3′-RNA cleavage, can lead to spikes of high signal intensities at the last nucleotide of exons or introns and at 3′-RNA cleavage sites. We computationally masked these nucleotide positions prior to peak calling to avoid any impact on subsequent analyses. PCR duplicates that can originate during the final amplification of the sequencing library and which can also lead to spikes of varying intensities were identified by unique molecular identifiers (UMIs) introduced during NET-seq library preparation. Moreover, UMIs also helped to identify reads that resulted from RT mispriming due to misalignment of the RT primer to adapter-like sequences within the nascent RNA (Figure 2A, (11)). We computationally removed these reads prior to peak calling.

Notably, we found that despite the usage of UMIs a substantial fraction (>30%) of RT mispriming events escaped detection. In order to avoid the formation of peaks from RT mispriming, we developed a new NET-seq protocol variant, termed nested NET-seq (Figure 2B). Nested NET-seq introduces two main changes to the NET-seq library preparation that were inspired by the HITS-CLIP approach (68). First, we designed a nested RT primer that lacked the first three nucleotides of the 3′-adapter. Second, we constructed a new reverse PCR primer that was complementary to the complete 3′-adapter sequence and contained a phosphorothioate bond protecting the final three nucleotides (Figure 2B). As a consequence only cDNAs with a complete 3′-adaptor sequence served as templates in the final PCR amplification and thus RT products from mispriming were not amplified.

The nested NET-seq replicates obtained from HeLa cells correlated well indicating the robustness of this approach (Figure 2C). The correlation between nested NET-seq and standard NET-seq data was high (Pearson’s coefficient, *R* > 0.8, Supplementary Figure S2). A main difference between nested NET-seq and standard NET-seq data was that the fraction of peaks with adapter-like sequences was strongly reduced (3.3–4 times less) in case of nested NET-seq (Figure 2D). This trend was also clearly visible at the single-gene level (Figure 2E). Together, these observations suggest that artifactual signal intensity peaks were systematically reduced in nested NET-seq data and that the remaining peaks reflect locations of high Pol II occupancy.

### A high-resolution map of genomic pausing sites

**Figure 3.**
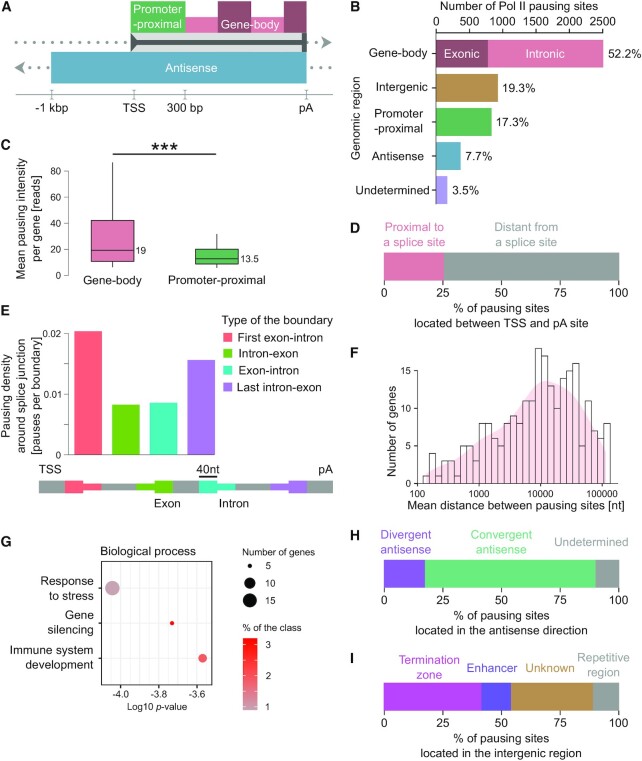
Pervasive transcriptional pausing in human cells. (**A**) Scheme of different genomic regions of interest. The direction of transcription is indicated by arrowheads. (**B**) Pausing site distribution over different genomic regions as defined in (A). (**C**) Mean nested NET-seq signal intensity per gene for pausing sites located in the gene-body or promoter-proximal region of low and medium expressed genes. The *P*-value was calculated by the Welch one-sided *t*-test. *P* = 0.00046 for the indicated comparison. Asterisks illustrate the significance. (**D**) Percentage of Pol II pausing sites located between the TSS and the pA site. The fraction of pausing locations in proximity to splice sites are in pink. (**E**) Distribution of splice site related pauses at distinct exon-intron junctions. The type of junction is color-coded and indicated by the scheme below. (**F**) Distribution of the mean distance between pausing sites per gene. (**G**) Gene ontology (GO) enrichment analysis for genes with a high pausing frequency. (**H**) Pause site distribution across genomic regions transcribed in the antisense direction by Pol II. (**I**) Pause site distribution across intergenic regions. (B–I) Data were obtained for HeLa cells.

We applied PDA to high-coverage nested NET-seq data generated for HeLa cells to obtain a high-resolution genomic map of Pol II transcriptional pausing sites in human cells. We called 4831 significant and high-confidence Pol lI occupancy peaks corresponding to potential Pol II pausing sites. The number of pausing sites was likely an underestimate due to the stringent criteria applied for peak calling and for statistical tests. About 75% and 20.6% of occupancy peaks were intra- (promoter-proximal, gene-body and convergent antisense) or intergenic (including divergent antisense), respectively (Figure 3A and B; Supplementary Figure S3A and Supplementary Table S3). About 3.5% of peaks could not be unambiguously assigned to a specific location and were not considered in further analyses. We first focused on the genic Pol II occupancy peaks and analyzed how these peak locations were distributed along genes (Figure 3A and Supplementary Figure S3A). We found that 17.3% of peaks were located in the promoter-proximal region of genes corresponding to promoter-proximal pauses (Figure 3A and B).

Notably, we found that the majority of Pol II pausing sites occurred outside of promoter-proximal regions, mainly throughout the gene-body (Figure 3B). The signal intensity of gene-body Pol II occupancy peaks was on average slightly higher as compared to corresponding peaks in promoter-proximal regions for low and medium expressed genes (TPM <100) (Figure 3C). This difference in pause intensity was not visible for highly expressed genes (TPM >100; Supplementary Figure S3B). About 31% and 69% of the gene-body pauses occurred over exons and introns, respectively (Figure 3B). Interestingly, a large fraction of Pol II peaks (25%) occurred in close proximity (±40 nt) to splice sites (Figure 3D). Pauses were especially prevalent at first exon-intron and last intron-exon junctions of genes (Figure 3E). These observations were consistent with the emerging view that transcriptional pausing is implicated in co-transcriptional splicing (27,69).

The average distance between consecutive pausing sites varied strongly between genes (Figure 3F). For the majority of genes, the distance was between 3000 and 30 000 nucleotides (nts). At both extremes we observed genes where Pol II pauses every 146 or 13 1994 nts, respectively. A gene ontology (GO) enrichment analysis revealed that genes with a higher pausing frequency were significantly enriched for the following biological processes: ‘response to stress’, ‘gene silencing’ and ‘immune system development’ (Figure 3G). This finding implies a potential role of frequent transcriptional pausing in these processes that needs to be further analyzed in future studies.

Given the prevalence of Pol II occupancy peaks throughout the gene-body region, we asked if they partially emerged from transcription initiation events at alternative promoters located within genes or from premature RNA cleavage and transcription termination. To address the first question, we re-analyzed NET-CAGE data (50) that provide a quantitative measure of TSSs at nucleotide resolution and that were available for HeLa cells. This analysis revealed no enrichment of TSSs upstream of gene-body Pol II occupancy peaks (Supplementary Figure S3C). To address the second question and to estimate potential premature RNA cleavage and termination sites, we re-analyzed RNA 3′ end-seq data upon RNA exosome (RRP40) depletion (52). RNA 3′ end-seq has been used to map 3′-ends of transcripts including unstable nascent transcripts (52). The re-analysis revealed that the vast majority of 3′-RNA ends mapped to canonical poly-adenylation (pA) sites at the 3′-end of genes (Supplementary Figure S3D). We also observed 3′-end signals at pausing locations in promoter-proximal regions (32% of all detected promoter-proximal pausing sites; Supplementary Figure S3D) that is consistent with prior observations of premature transcription termination in this region (70). We detected 3′-end signals for a subset of gene-body pausing sites (16% of all detected gene-body pausing sites; Supplementary Figure S3D). These percentages are likely overestimates of potential premature termination since part of the 3′-end signals stem from nascent 3′-RNA ends of Pol II elongation complexes and in these cases are not indicative for premature termination. Moreover, poly-adenylation signals (PASs; (71)) were not enriched in proximity to gene-body pausing sites (Supplementary Figure S3E). These findings suggest that the vast majority of observed gene-body Pol II peaks are most likely not linked to intra-genic initiation or premature RNA cleavage and termination events.

Since NET-seq provides DNA strand-specific Pol II occupancies (42), this analysis revealed significant Pol II occupancy peaks in antisense direction (Figure 3B). Although we detected Pol II pausing during divergent antisense transcription upstream and in the opposite orientation of protein-coding and long non-coding RNA (lncRNA) genes (Figure 3H and Supplementary Figure S3A), the majority of antisense pauses were detected within gene-body regions, previously termed convergent antisense transcription (11) (Figure 3H and Supplementary Figure S3A). This suggests that antisense transcription, similarly to transcription in the sense direction, is punctuated by pauses in human cells.

PDA also uncovered a large fraction of potential pausing sites in intergenic regions. The majority of these peaks were located at enhancers, the termination zone (the region where transcription usually terminates (31,72)) and at repetitive genomic regions (Figure 3I). A set of peaks occurred at non-annotated intergenic regions (Figure 3I).

We extended our analysis to other human cell types for which high-coverage NET-seq data were available including MOLT4 (66), HEK293T (11) and K562 cells (73). These analyses revealed that the distribution of called peaks along different genomic regions was very similar as for HeLa cells (nested NET-seq), with the majority of peaks located outside of the promoter-proximal region (Figure 3A and B; Supplementary Figure S3F). However, conclusions drawn from these comparative analyses with non-nested NET-seq data need to be done cautiously due to their inflitration with RT mispriming artifacts.

Together, these data suggest widespread Pol II pausing during transcription in human cells and are not restricted to promoter-proximal regions or to transcription in the sense direction.

### DNA-sequence properties underlie pervasive pausing

**Figure 4.**
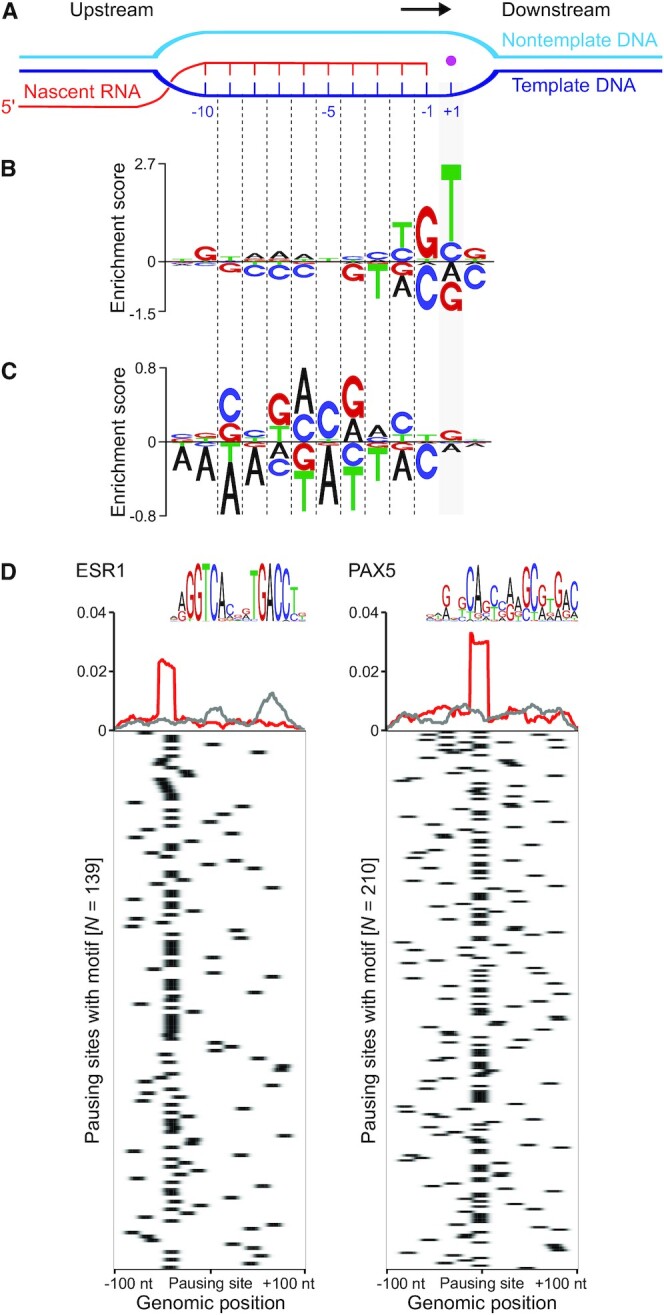
*De novo* pausing motif discovery and sequence analysis. (**A**) Schematic view of the transcription bubble. The pink dot corresponds to a Mg^2+^ ion marking the active site of Pol II. -1 refers to the last nucleotide of the nascent RNA. +1 indicates the position in the DNA template where the next incoming NTP binds. The direction of transcription is indicated by a black arrow. This model is based on recent evidence from structural studies indicating that the RNA-DNA hybrid that spans the active site of the mammalian Pol II elongation complex is 9–10 bp long (101). (**B** and**C**) Enrichment logos for promoter-proximal pause (B) and gene-body pause sites (C). (**D**) Transcription factor binding motifs enriched in proximity to a set of gene-body pauses. Metaplot and heatmap of the binding motifs for transcription factors ESR1 and PAX5 are shown for pausing (red) and non-pausing sites (gray). The corresponding sequence motif is shown above the metaplot.

What are potential determinants of pervasive transcriptional pausing in human cells? To address this question, we first investigated whether distinct DNA sequences were enriched at or in close proximity to pausing sites. A main advantage of NET-seq over ChIP-seq data was the high spatial resolution (Figure 1A and B). This allowed us to precisely extract the DNA sequences underlying Pol II pause positions and to search for common sequence motifs. For motif discovery, we first focused our analysis on promoter-proximal pauses. Notably, this analysis uncovered a new DNA motif that was significantly enriched at promoter-proximal Pol II pausing sites (Figure 4A and B). The motif with the DNA consensus sequence G_-10_Y_-2_G_-1_Y_+1_, where Y is a pyrimidine, differed from known *cis*-motifs implicated in promoter-proximal pausing in Drosophila and mammalian cells (Supplementary Figure S4A). The motif consists of two parts: the G_-10_ at the upstream fork junction of the RNA-DNA hybrid and the Y_-2_G_-1_Y_+1_ region spanning the active site of Pol II and the downstream fork junction of the RNA-DNA hybrid.

We extended the DNA sequence analysis to pausing sites throughout the gene-body. Although this analysis did not recover a clear motif, we identified a DNA signature that was significantly linked with widespread Pol II pausing at gene-body regions, termed the gene-body pause signature (GPS) (Figure 4C). This pause signature differed from sequences that have been previously ascribed to pausing in different model systems and showed no similarities with known transcription factor binding sites. For the identification of GPS, the nested NET-seq approach was crucial. When we applied PDA on standard NET-seq data, for which it is not possible to systematically account for RT mispriming events, an artifactual sequence motif was retrieved that resembled the adapter sequence used in the library preparation (Supplementary Figure S4B). This illustrates the importance of removing or ideally avoiding RT mispriming artifacts prior to sequence analyses especially in the context of high-resolution occupancy profiling studies.

Previously, we found that Pol II can accumulate upstream and downstream of distinct transcription factor binding sites (TFBSs) located throughout the gene-body region (11). Therefore, we studied whether specific TFBSs were enriched in close proximity to Pol II pausing locations. From 639 TFs for which TFBSs were available and that were included in our analysis, we found a significant enrichment of TFBSs for ESR1 and PAX5 at a subset of gene-body pauses (Figure 4D). Both factors were previously linked to Pol II transcription (74,75). These findings suggest a potential role of distinct *trans*-acting transcription factors in Pol II pausing throughout gene-body regions. To further explore the molecular mechanisms of gene-body pausing regulation by different transcriptions factors represent an interesting subject for future investigations. Together, these results suggest that DNA-sequences underlie pervasive transcriptional pausing in human cells, and also imply potential contributory roles of *trans*-acting factors.

### Machine learning model reveals pausing determinants

**Figure 5.**
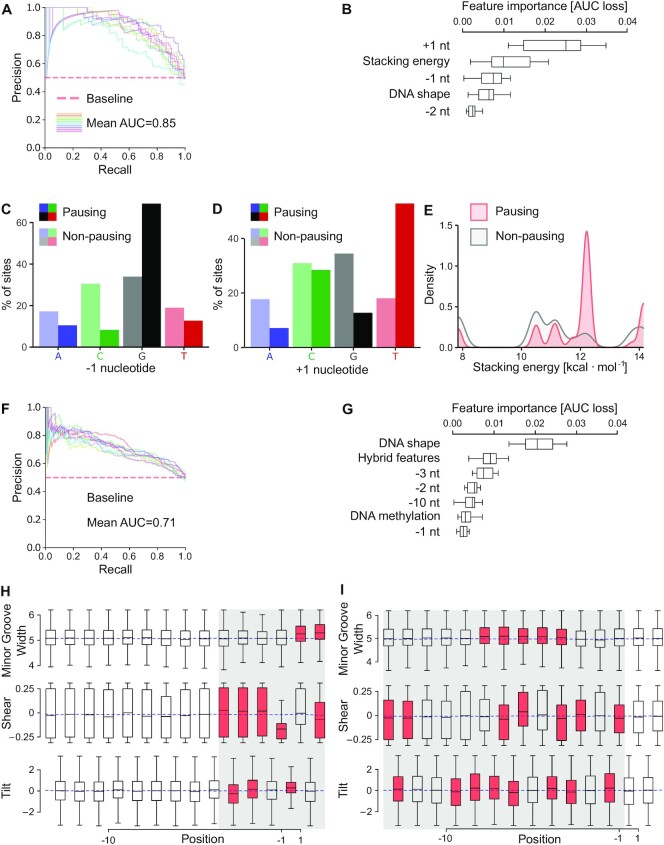
Machine learning model identifies determinants for pervasive transcriptional pausing. (**A** and**F**) Precision recall curve derived from 10-fold cross-validation of classification model for pauses in the promoter-proximal (A) and in the gene-body (F) region. (**B**) Features important for the classification determined by permutation importance values for promoter-proximal pauses. Only features with permutation importance significantly greater than zero are shown. (**C**) Nucleotide distribution at the -1 position for promoter-proximal pausing and non-pausing sites. (**D**) Nucleotide distribution at the +1 position for promoter-proximal pausing and non-pausing sites. (**E**) Distribution of stacking energy between bases of neighboring nucleotides at positions -1 and +1 of promoter-proximal pausing sites and of locations with no pauses. (**G**) Features important for the classification as in (B) but for gene-body Pol II pauses. (**H** and**I**) Boxplots of selected DNA shape features (Minor Groove Width, Shear and Tilt) plotted with mean value for promoter-proximal (H) and gene-body (I) pausing sites. The mean of the distribution of the DNA shape features obtained for non-pausing sites is visualized by a blue dashed line. Positions where the *P*-value is <10^−8^ (Welch’s *t*-test, Bonferroni corrected) are indicated in red. DNAshapeR (56) was used for feature predictions.

Given that DNA sequence properties underlie pervasive pausing, we asked whether we could use this knowledge to predict the pausing potential for each nucleotide along genes. To address this question, we created a machine learning model for transcriptional pausing that was based on a random forest classifier. We first focused our modeling on promoter-proximal Pol II pauses. We tested whether we could predict promoter-proximal pauses from DNA sequence features genome-wide. Notably, our model predicted pausing sites with high accuracy assessed by 10-fold cross-validation (Figure 5A). In addition to its predictive value, this approach had two main advantages. First, it informs on the importance of each feature for pause prediction that we included into the model (Figure 5B). Second, we could add additional features to the model and test its predictive value for pausing sites allowing the identification of new pause determinants. An exhaustive list of features included in this analysis is provided in Supplementary Table S1.

We found that among the most important predictors for Pol II pausing in the promoter-proximal region were nucleotides at positions +1 (insertion site for incoming NTP) and -1 (last nucleotide of the RNA-DNA hybrid) of the DNA template strand (Figure 4A and Figure 5B). Further analyses revealed a preference for a G or T nucleotide at positions -1 or +1, respectively (Figure 5C and D). This finding was consistent with the uncovered promoter-proximal pause motif (Figure 4B). An important predictor for pausing was also the stacking energy between bases (Figure 5B). Additional analyses showed that the stacking energy between bases of the DNA was increased at promoter-proximal pausing sites (Figure 5E). Moreover, the DNA shape and the identity of the -2 nucleotide were predictive for promoter-proximal pausing.

We next performed machine learning on gene-body pauses to investigate whether they were determined by similar or distinct features as compared to pauses in the promoter-proximal region. Our approach could predict gene-body pauses, although at a lower accuracy as compared to promoter-proximal pauses (Figure 5F). Interestingly, this analysis revealed the DNA shape as the most predictive sequence feature for gene-body pauses (Figure 5G). Moreover, several characteristics of the RNA-DNA hybrid emerged as strong predictors (Figure 5G). Similarly to promoter-proximal pauses, the -1 and -2 nucleotides of the DNA template strand were identified as predictors. However, in case of gene-body pauses additional positions of the DNA template strand upstream of the active site including positions -3 and -10 were predictive for pausing (Figure 5G). DNA methylation was also predictive for gene-body but not promoter-proximal pauses (Figure 5B–G).

Given our finding that the DNA shape was predictive for widespread gene-body pauses and to a less extent also for promoter-proximal pauses, we further characterized the contribution of DNA shape features in Pol II pausing. Interestingly, this analysis revealed that several DNA shape characteristics were linked to widespread pausing in promoter-proximal and gene-body regions (Figure 5H–I; Supplementary Figure S5A and B). For promoter-proximal pausing the shape of the DNA encompassing the downstream fork junction of the RNA-DNA hybrid and of the downstream DNA was critical (Figure 5H and Supplementary Figure S5A) whereas for gene-body pauses the structural features of the central region of the RNA-DNA hybrid and of the upstream DNA were crucial (Figure 5I and Supplementary Figure S5B). For instance, the ability for tilting between neighboring base pairs as well as the minor groove width was significantly increased at promoter-proximal or gene-body pausing locations (Figure 5H and I). The finding that DNA shape plays a role in sequence-induced pausing extends previous observations obtained for promoter-proximal pauses (76,77) to widespread Pol II pausing in human cells. Together, these studies indicate the importance of DNA sequence properties in pervasive pause determination and suggest that Pol II pausing is a multifactor-dependent process in human cells. This analysis also reveals differences in determinants for promoter-proximal and gene-body pausing.

### Sequence determinants of transcriptional pausing are conserved

We next asked whether the sequence determinants of pervasive RNA polymerase pausing were evolutionarily conserved. To address this question, we re-analyzed available NET-seq data for bacteria (*Escherichia coli*) (23), budding yeast (*Saccharomyces cerevisiae*) (78) and plants (*A. thaliana*) (37). We used PDA to determine the pausing landscapes of RNA polymerase in bacteria and for Pol II in yeast and plants at nucleotide resolution (Supplementary Tables S4–S6). Next, we employed our modeling approach to reveal determinants for all pauses within genes since clear evidence for promoter-proximal pausing has not yet emerged in these organisms (79,80).

**Figure 6.**
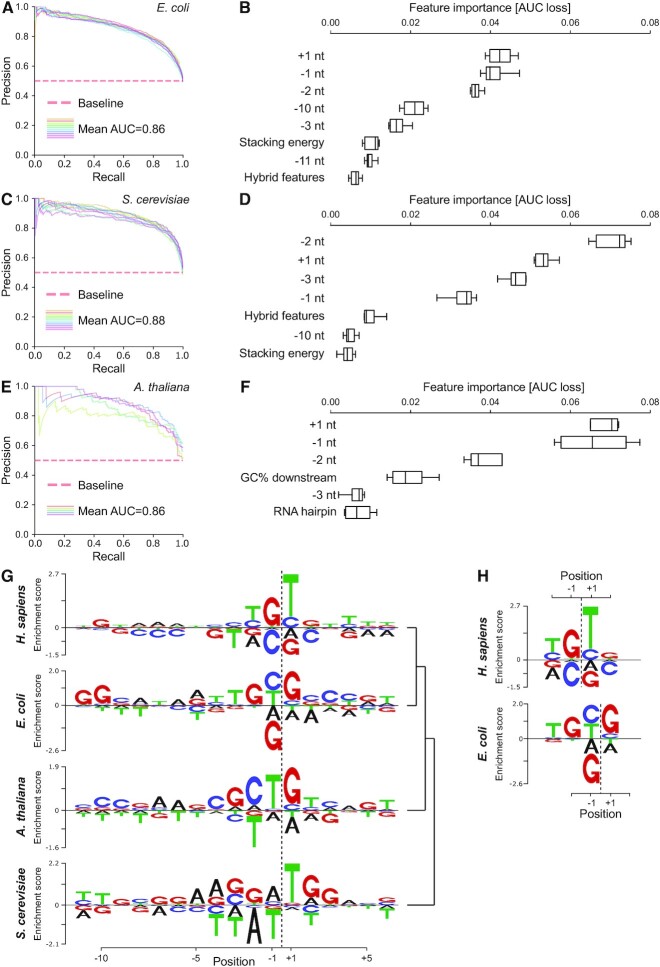
Sequence determinants for pervasive transcriptional pausing are conserved. (**A, C** and**E**) Precision recall curves derived from cross-validation of classification model for *E. coli* (A), *S. cerevisiae* (C) and *A. thaliana* (E). (**B, D** and**F**) Features important for the classification for *E. coli* (B), *S. cerevisiae* (D) and *A. thaliana* (F). Only features with permutation importance value significantly greater than zero are shown. (A–F) All pauses within genes are included. (**G**) Enrichment logos for *H. sapiens* (pauses in promoter-proximal region), *E. coli* (pauses within gene), *S. cerevisiae* (pauses within gene) and *A. thaliana* (pauses within gene). Similarity between logos is shown (right). Clustering of the logos was obtained using cosine similarity. (**H**) Comparison of enrichment logos encompassing the downstream RNA-DNA hybrid fork junction derived for promoter-proximal Pol II pauses of *H. sapiens* and for widespread pauses of *E. coli*.

We first focused on *E. coli* since it was shown that RNA polymerase pauses ubiquitously during transcription in bacteria (19,20). Our modeling approach predicted pauses with high accuracy (Figure 6A). Notably, this analysis revealed sequence features as strong predictors for pausing that strongly overlapped with those for human cells (Figures 5B and 6B). Important determinants for ubiquitous pausing in bacteria were distinct nucleotide positions in the DNA template especially at both ends of the RNA-DNA hybrid region (-10/-11 and -2/-1/+1 positions) (Figure 6B). This is in line with previous studies in bacteria (21–23,81,82).

We extended these analyses to Pol II pausing in budding yeast and plants. Machine learning modeling also predicted pausing in yeast and plants (Figure 6C and E) and revealed pausing determinants that strongly overlapped with those of human and bacterial cells (Figure 6D and F). For all species analyzed in this study, the -2/-1 and +1 positions in the DNA template spanning the active site of RNA polymerase and the downstream fork junction of the RNA-DNA hybrid had the highest predictive value emerging as the main sequence-based pause determinants.

Given our finding that key sequence determinants of pausing were conserved from bacteria to humans, we next investigated whether this also applied to the DNA consensus sequence that we uncovered for promoter-proximal pausing in human cells (Figures 4B and 6G). These analyses retrieved sequence motifs underlying pausing in *E. coli*, *S. cerevisiae* and *A. thaliana* (Figure 6G). Interestingly, all motifs overlapped with the region of the downstream fork junction of the RNA-DNA hybrid indicating its universal importance for sequence-induced pausing. The consensus pause sequence obtained for *E. coli* was almost identical with a previously described pause motif for bacteria (21–23,81,82) (Supplementary Figure S6A).

A comparison of the uncovered motifs for the different species revealed striking similarities (Figure 6G). Notably, the sequence motif obtained for bacteria was highly similar to the motif underlying promoter-proximal pauses in human cells (Figure 6G). The bacterial pause motif was shifted upstream by a single nucleotide as compared to the human consensus pause sequence (Figure 6H). Similarities of the human and bacterial pause motifs with the corresponding consensus sequences from plants and yeast could also be detected for the portion spanning the active site of RNA polymerase (Figure 6G). Interestingly, for all species investigated here the consensus pause motif contained a ‘TG’ dinucleotide at the active site region of RNA polymerase. In the case of humans and bacteria this dinucleotide is even repeated. This finding is consistent with a previous *in vitro* study showing that a TG dinucleotide motif can provoke a slowdown of transcribing bacterial RNA polymerase and that a repeat of this motif (TGTG) had an even stronger effect on pause induction (83). This motif also has interesting similarities with a pause stabilizing element (Inr-G) in Drosophila which contains a ‘GT’ dinucleotide (84).

However, the similarity of the plant and yeast pause motifs with those of human and bacteria was generally less pronounced. We cannot rule out that the latter observation was partially due to the lower sequencing depth of NET-seq data available for plants and yeast that led to fewer detected Pol II pausing sites. Together, these data show that key determinants of RNA polymerase pausing are conserved from bacteria to humans suggesting a universal DNA sequence-based molecular mechanism underlying pervasive pausing across the tree of life.

## DISCUSSION

Here, we developed an algorithm for robust peak detection from single-nucleotide resolution profiling data and a new NET-seq protocol to investigate DNA sequence-induced transcriptional pausing in human cells. Our study shows that common artifacts originating from several steps during sequencing library preparation can have a strong impact on data interpretation. As a main source for artifactual peaks commonly misinterpreted as Pol II occupancy peaks and potential pausing sites we identified RT mispriming. Although the usage of UMIs can help to computationally identify reads from PCR duplication and RT mispriming, our studies also reveal that a large fraction of mispriming events usually escape detection and cannot be removed computationally. By developing and applying nested NET-seq, we show that artifactual signal intensity peaks are strongly reduced mainly by avoiding the amplification of RT mispriming products. RT mispriming represents a common source of artifacts in other widely used genome-wide and transcriptome-wide profiling approaches including precision nuclear run-on sequencing (PRO-seq), global run-on sequencing (GRO-seq), ChIP-seq and RNA-seq (11,39–41,67,68). These findings have therefore broad implications for the interpretation of data obtained from previous genome- and transcriptome-wide studies that use RT during library preparation.

The transcriptional pausing landscape of Pol II in human cells is more diverse than originally anticipated. The majority of Pol II pausing sites that we have detected are located outside of promoter-proximal gene regions. A large fraction of these pauses are distributed along the gene-body. The frequency of pausing sites along the gene-body strongly varies between genes. We found active genes with no or only one high-confident pausing site and genes with pauses every 140 nts. The latter observation suggests that transcription elongation by Pol II can be very discontinuous at a large set of genes where productive elongation is frequently interrupted by pauses. A subset of these gene-body pauses are strategically positioned at splice junctions and especially at the first exon-intron and the last intron-exon boundary. These observations are consistent with the emerging view that pausing is required for co-transcriptional splicing (27,69). Pausing across the gene-body is not restricted to the sense direction but is also prevalent throughout antisense transcription. If antisense pausing interferes with sense transcription will be an interesting subject for future analyses.

Are gene-body pauses distinct from pauses in promoter-proximal gene regions? Interestingly, a large set of promoter-proximal and gene-body pauses occur non-randomly at the same genomic nucleotide position in biological replicate measurements. Moreover, distinct DNA sequence properties such as the nucleotide composition and the DNA shape are significantly linked with both types of pauses, and we also show that pausing sites can be predicted with high probability from DNA sequence features alone. These findings illustrate the importance of DNA sequence characteristics in the control of both promoter-proximal and gene-body pauses in human cells. In addition, the signal intensities of pauses in the promoter-proximal and gene-body regions are on average similar. Despite these similarities, we also observe differences between both classes of pauses. First, the DNA sequence underlying both types of pauses differs. Whereas a clear sequence motif was uncovered for pauses in the promoter-proximal region, no *cis*-element could be retrieved for gene-body pauses. Second, the predictability of gene-body pauses is lower as for promoter-proximal pauses. Third, RNA-DNA hybrid features and characteristics of the upstream DNA are more critical determinants for gene-body pauses as compared to promoter-proximal pauses. Moreover, DNA methylation is only linked to a set of gene-body pauses. These differences support the idea that gene-body pauses are distinct and more heterogeneous as promoter-proximal pauses that usually occur within a narrow region downstream of the transcription start site.

In this study, we uncovered a new sequence motif that underlies widespread promoter-proximal pausing in human cells. Although the position of the motif within the DNA template strand overlapping with the downstream fork junction of the RNA-DNA hybrid is consistent with recent studies, the nucleotide sequence differs strongly from sequence elements that have been implicated in promoter-proximal pausing (24–26). Likely explanations for this difference are the high spatial resolution of pause site detection that allowed us to precisely extract the underlying DNA sequence also of closely spaced pausing peaks, and sequence context based normalization. The latter was critical to minimize sequence biases originating from the high GC content in promoter-proximal gene regions (85). Interestingly, the promoter-proximal pause motif shows similarities to the consensus sequence of the following core promoter elements located downstream of the TSS (86,87): the downstream core promoter element (DPE) (88), the downstream core element (DCE) (89,90) and the recently uncovered human DPR core promoter element (91) (Supplementary Figure S6B). The region where these *cis*-elements are located strongly overlap with the region where promoter-proximal pausing of Pol II usually occurs. These findings extend previous observations that have linked core promoter elements with transcriptional pausing in Drosophila (12,24,84).

Widespread transcriptional pausing of Pol II in human cells has apparent similarities to RNA polymerase pausing in bacteria. First, the pervasive nature of pausing is similar between Pol II and bacterial RNA polymerase. Similarly to bacteria (21–23,92), pausing in human cells occurs throughout the transcribed region and is not restricted to promoter-proximal gene regions. Moreover, the finding that Pol II pauses frequently during antisense transcription is consistent with observations in bacteria (21). Second, sequence-dependent features of pausing in bacteria strongly overlap with determinants of widespread Pol II pausing. Nucleotide positions at both ends of the RNA-DNA hybrid region (-10 and +1/-1/-2/-3) are critical for pausing of Pol II as well as for widespread pausing in *E. coli*. These findings are in line with previous observations in bacteria providing evidence that the upstream and downstream fork junctions of the RNA-DNA hybrid are critical for pause induction (21–23,81,82). Notably, the sequence motif G_-10_Y_-2_G_-1_Y_+1_ uncovered for Pol II promoter-proximal pausing has striking similarities with the bacterial pause motif G_-10_Y_-3_G_-2_Y_-1_G_+1_ with one main difference. The Y_-2_G_-1_Y_+1_ portion of the Pol II promoter-proximal pause motif spanning the active site is shifted downstream by a single nucleotide as compared to the bacterial consensus pause sequence. A potential explanation for this difference can be found in the oscillating behavior of the elongation complex oscillating in a thermal equilibrium by one nucleotide position between pre- and post-translocated states (93). It can be that human Pol II and bacterial RNA polymerase were preferentially captured in the post- or pre-translocated state, respectively. The bacterial pause motif that we retrieved in this study is almost identical with the pause signal described recently for bacteria (21–23,81,82). The similarities of transcriptional pausing in human and bacterial cells are also consistent with the similar 3D architecture of the active site between Pol II and bacterial RNA polymerase, and with the conserved catalytic mechanism (94,95). Altogether, these similarities point to a universally conserved sequence-dependent transcriptional pausing mechanism.

Our findings converge on the following model of frequent DNA sequence-induced transcriptional pausing and of its potential roles in human cells. As Pol II translocates along the DNA template, multiple DNA sequence properties, rather than a single feature alone, can provoke RNA polymerase to halt at distinct nucleotide positions genome-wide. These widespread punctuations of transcription elongation represent potential points for regulation (27,96). In bacteria, ubiquitous pausing can couple transcription with protein synthesis (23,97). Therefore, it is tempting to speculate that gene-body pausing in human cells can create regulatory opportunities at later stages of transcription to link Pol II transcription with other processes including RNA processing and DNA repair. The observation that a large set of gene-body pauses occur at strategic locations, such as at intron-exon boundaries, supports this idea. This view is also in line with the kinetic coupling model according to which slow elongation and pausing can create ‘windows of opportunity’ to link other processes such as RNA splicing to transcription and to enable their co-transcriptional control (98).

We expect that future studies will identify new molecular mechanisms that control widespread pausing outside of promoter-proximal gene regions in human cells including potential functions of *trans*-acting factors. Given the recent implications of a change in promoter-proximal pausing and transcription elongation in human pathology (13,66,99,100), an interesting future direction will be to explore potential contributory roles from altered gene-body pausing patterns.

## DATA AVAILABILITY

Scripts for data processing and analysis are accessible through our GitHub site: https://github.molgen.mpg.de/MayerGroup/Pervasive_PolII_pausing. All sequencing data (NET-seq and nested NET-seq) are available at GEO with the accession code: GSE162857.

## Supplementary Material

gkab208_Supplemental_Files
