# Supplementary material for: Conserved DNA sequence features underlie pervasive RNA polymerase pausing
Source: Nucleic Acids Res. 2021 Mar 31;49(8):4402–20. doi: 10.1093/nar/gkab208 (PMC8096220; doi:10.1093/nar/gkab208)
Supplement: gkab208_Supplemental_Files [file gkab208_supplemental_files.zip › Supplement_Gajos_Mayer.pdf]

SUPPLEMENTAL TABLE AND FIGURE LEGENDS

Supplemental Figure 1. (A) Venn diagram showing the overlap of significant peaks detected for biological NET-seq replicates obtained for HeLa S3 cells (11). **(B)** Heatmap showing the percentage of significant peaks detected for NET-seq datasets co-occurring at the same position in human HeLa S3 (11), HEK293T (11) and MOLT4 (66). **(C)** Relationship between the sequencing depth of NET-seq data and number of called peaks. A reduction in the sequencing depth was obtained by random subsampling of raw reads.

Supplemental Figure 2. Correlation analysis of biological replicate data sets obtained by standard or nested NET-seq measurements. The Pearson correlation coefficient (*R*) as indicated was derived from the Pol II occupancy of genes. TPM: Transcripts Per Million.

Supplemental Figure 3. (A) Scheme of different genomic regions with an emphasis on different antisense regions. Divergent antisense is the region upstream of the TSS and in opposite orientation to an annotated gene. Convergent antisense spans the region in opposite orientation to an annotated gene (from TSS to pA site). **(B)** Mean nested NET-seq signal intensity per gene for pausing sites located in the gene-body or promoter-proximal region of highly expressed genes (TPM >100). The *p*-value was calculated by the Welch one-sided *t*-test. ns: not significant **(C)** Boxplot quantification of average NET-CAGE signal density upstream of (i) random positions within the gene-body, (ii) pausing sites within the gene-body, and (iii) pausing sites in the promoter-proximal region. Significance of differences was calculated by a Welch *t*-test and significant differences in mean NET-CAGE signal density are indicated by asterisks. The calculated *p*-values for the comparisons between random positions and promoter-proximal sites, and between pausing sites within the gene-body and promoter-proximal sites were 0.013 or 0.0076, respectively. ns: not significant. **(D)** Boxplot quantification of average RNA 3' end-seq signal density around (i) random positions within the gene-body, (ii) pausing sites within the gene-body, (iii) pausing sites in the promoter-proximal region, (iv) pA sites. RNA 3' end-seq data obtained for HeLa cells was re-analyzed from (52). **(E)** Metaplots of polyadenylation signals in proximity of pausing (red), non-pausing (grey) and polyadenylation sites (grey). The corresponding sequence motif is shown above the metaplot. Polyadenylation signals were based on (71). **(F)** Pausing site distribution over different genomic regions for HEK293T (NET-seq) (11), MOLT4 (NET-seq) (66) and K562 (mNET-seq) (73) cells.

Supplemental Figure 4. (A) Known pausing motifs for *Drosophila melanogaster* (*D. melanogaster*) and *Homo sapiens* (*H. sapiens*). The pausing motif of *D. melanogaster* (top row) is also known as the ‘pause button’ (24). The exact location of the pause button in relation to the Pol II active site is unclear. Both human pause motifs were aligned based on

their location regarding the Pol II active site. A scheme of the transcription bubble is shown (last row). The scheme uses the same color-code as in Figure 4A. The original references for pause motif discovery are given (right column). **(B)** Artifactual sequence motif retrieved upon peak calling from standard NET-seq data. The motif resembles the adapter sequence used during the standard NET-seq library preparation and is located downstream of the peak.

Supplemental Figure 5. (A,B) Boxplots of selected DNA shape features (Buckle, Opening, Propeller Twist, Stagger, Stretch, Helix Twist, Rise, Roll, Shift, and Slide) plotted with mean value for promoter-proximal **(A)** and gene-body **(B)** pausing sites. The mean of the distribution of DNA shape features obtained for non-pausing sites is visualized by a blue dashed line. DNAshapeR (56) was used for feature predictions. Boxes at nucleotide positions where the p-value is $<10^{-8}$ (Welch's *t*-test, Bonferoni corrected) are in red. -1 refers to the nucleotide in the DNA template strand that matches with the 3'-end of the nascent RNA. +1 indicates the position in the DNA template where the next incoming NTP binds.

Supplemental Figure 6. (A) Comparison of pausing motifs obtained for *E. coli.* The pausing motif was derived from a re-analysis of NET-seq data available for *E. coli* (top row) (23). The pausing motif as revealed previously (23) is shown below. **(B)** Comparison of the promoter-proximal Pol II pausing motif (top row) with core promoter elements (following rows). For the downstream core promoter element (DPE) and the DPR core promoter element consensus DNA sequences are shown whereas for the downstream core element (DCE) II DNA sequence only is shown.

Supplemental Table 1. List of features included in machine learning models.

Supplemental Table 2. Parameters of the random forest classifiers.

Supplemental Table 3. Genomic coordinates of high-confidence Pol II pausing sites for HeLa S3 cells.

Supplemental Table 4. Genomic coordinates of high-confidence RNA polymerase pausing sites for *E. coli.*

Supplemental Table 5. Genomic coordinates of high-confidence Pol II pausing sites for *S. cerevisiae.*

Supplemental Table 6. Genomic coordinates of high-confidence Pol II pausing sites for *A. thaliana.*

Supplemental Figure 1

A

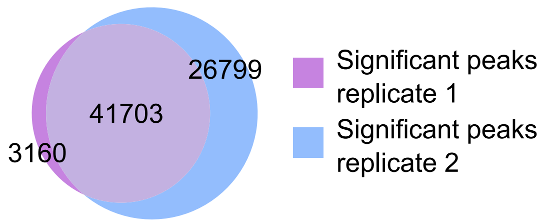

B

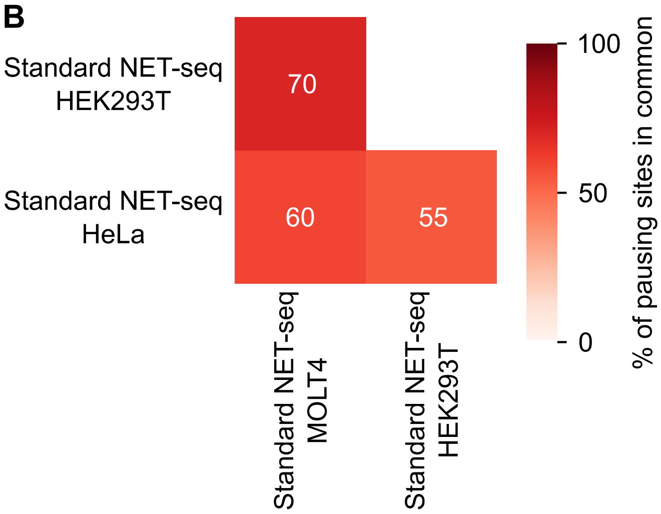

C

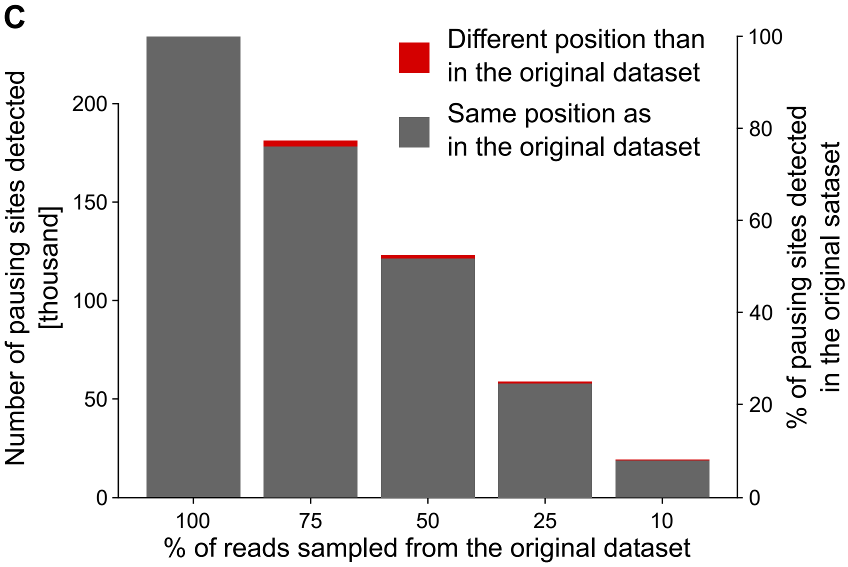

Supplemental Figure 2

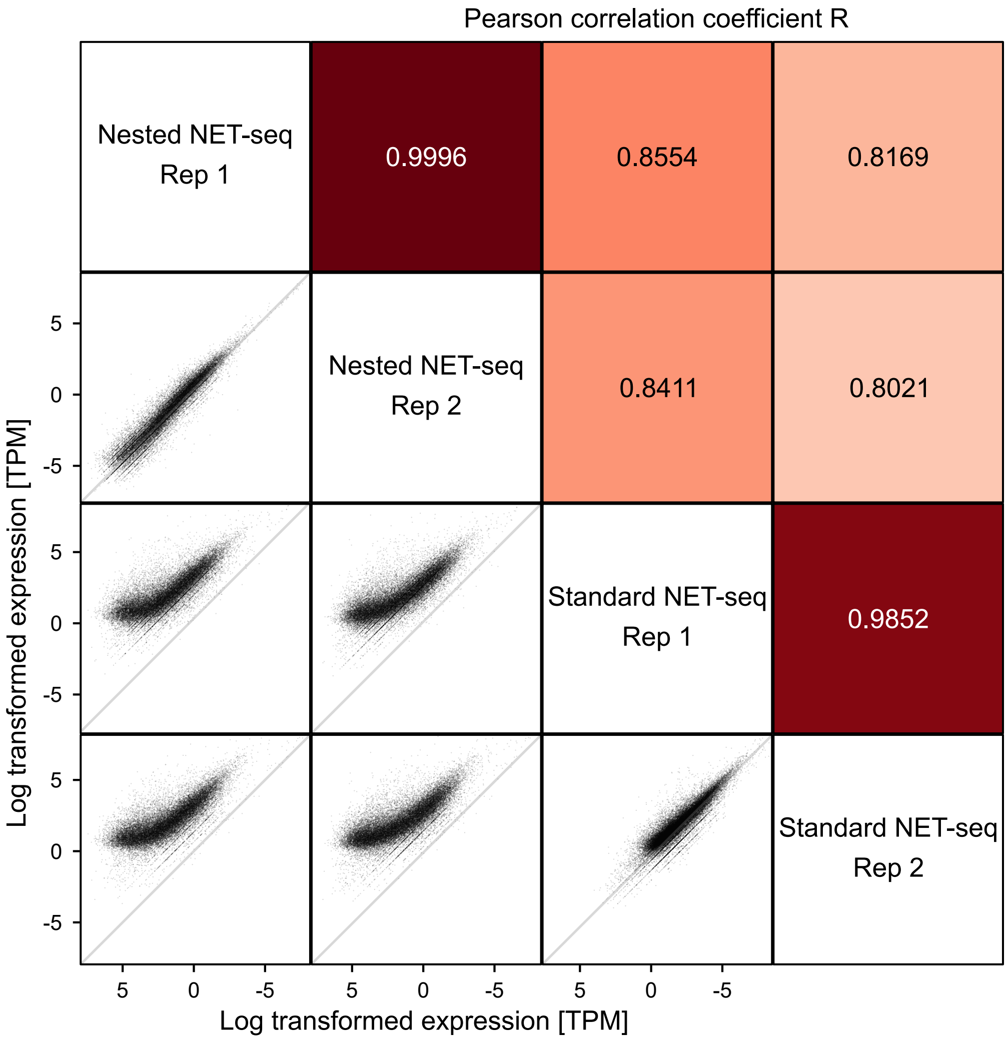

Supplemental Figure 3

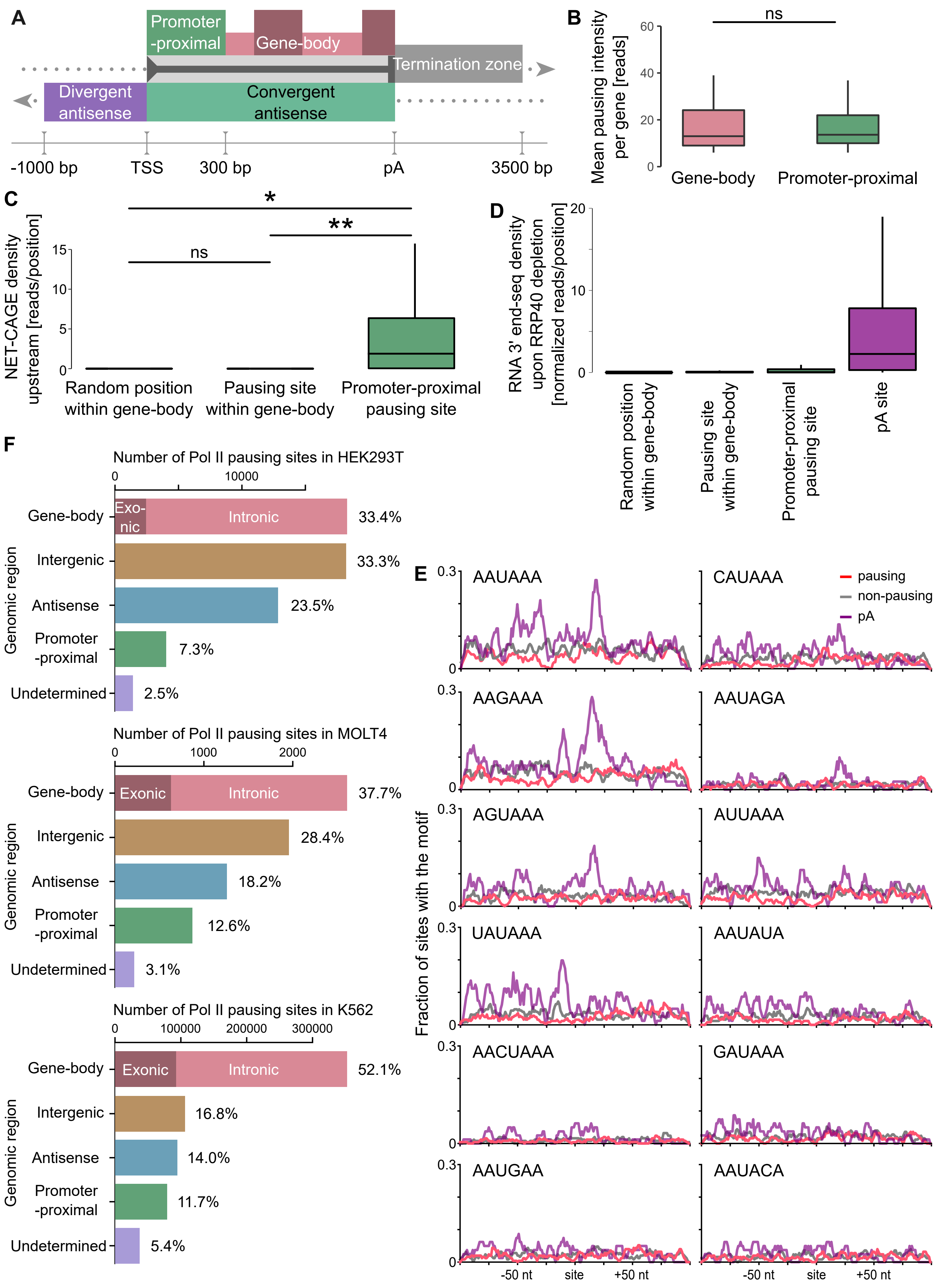

Supplemental Figure 4

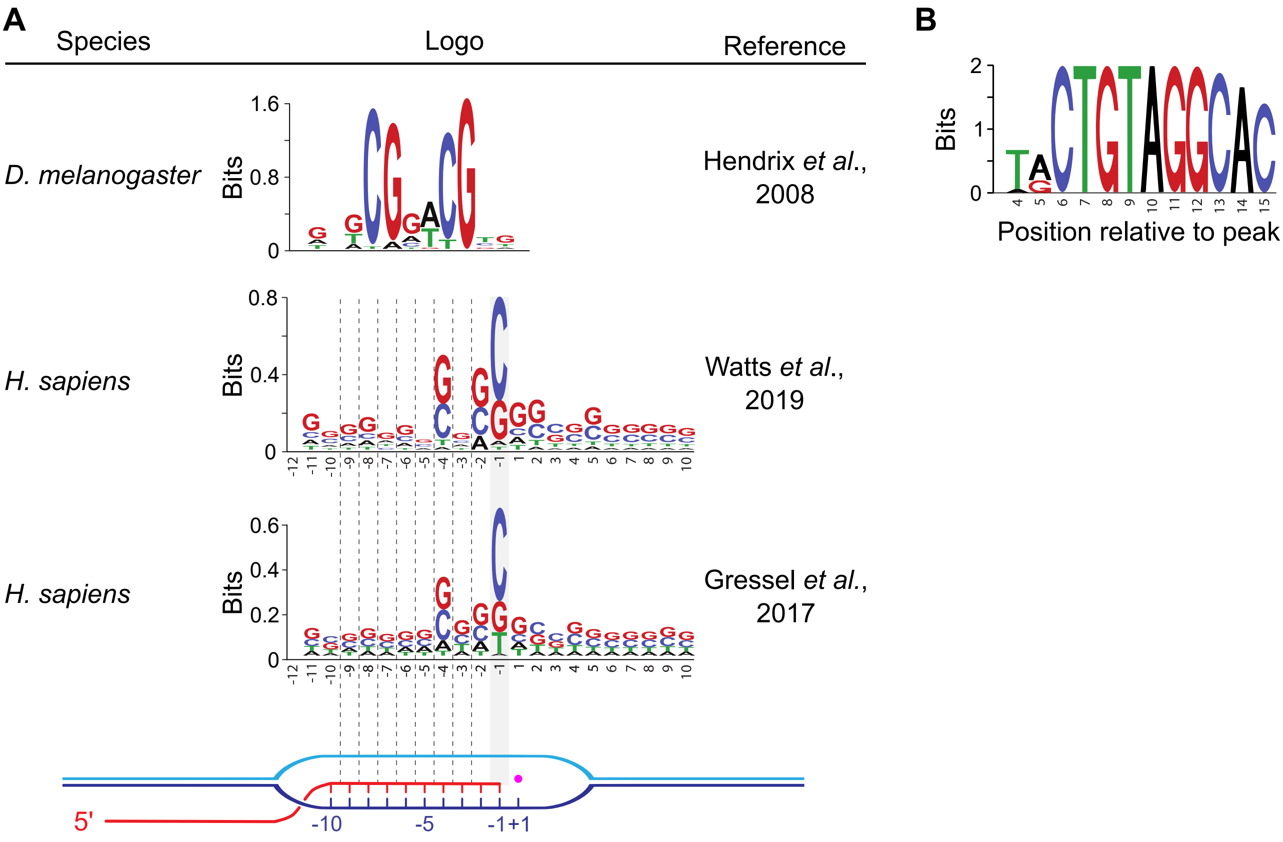

Supplemental Figure 5

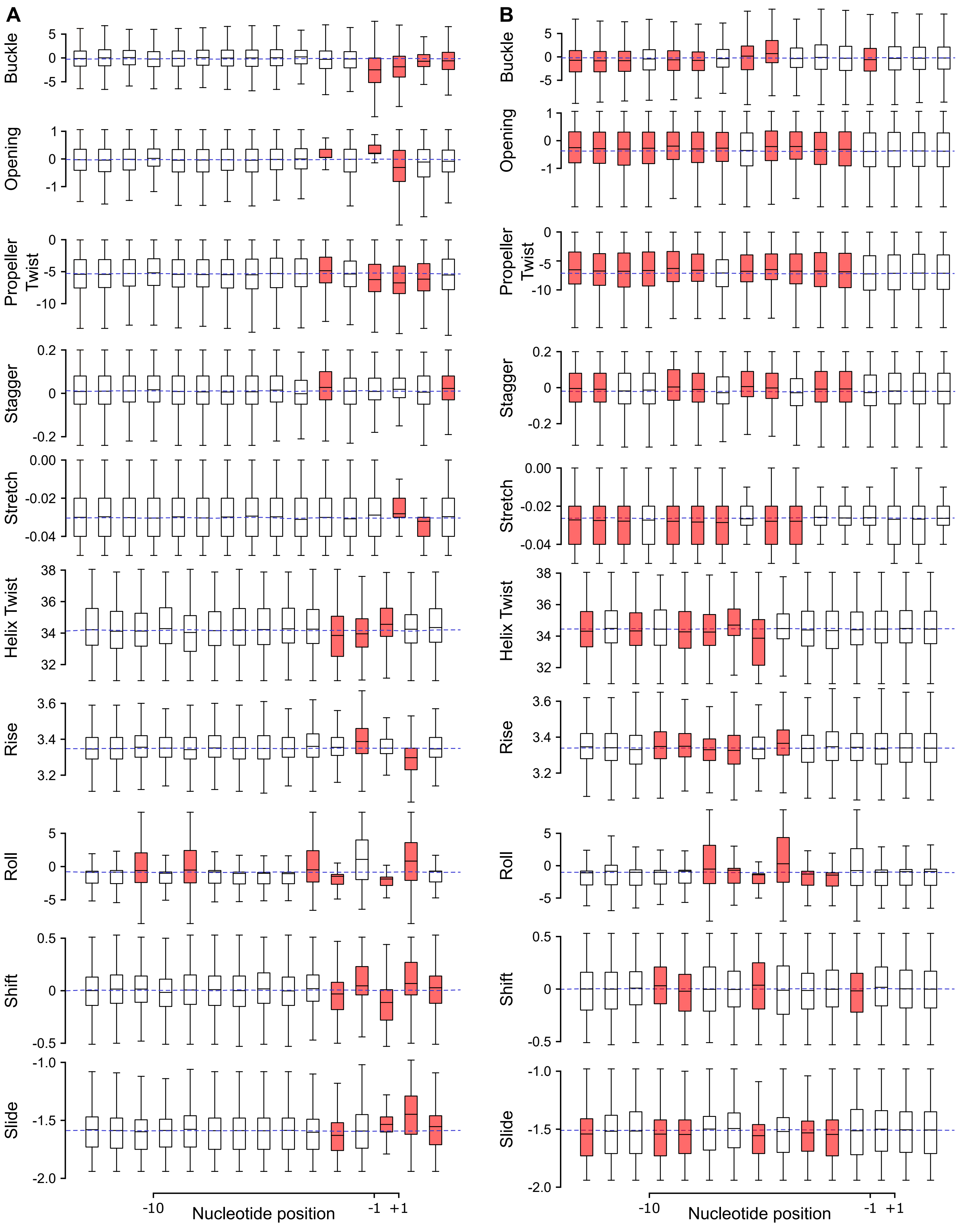

Supplemental Figure 6

A

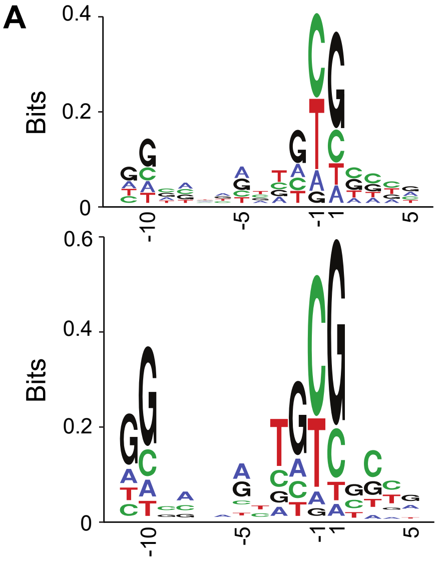

B

Promoter-proximal pause motif

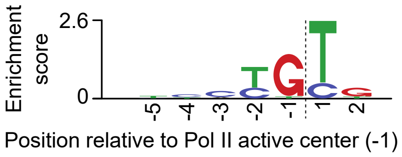

**Promoter element**	**Position relative to TSS**	**Sequence/Logo**
DCE II	16-21	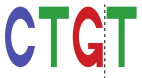
DPE	28-34	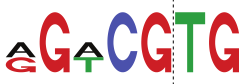
DPR (downstream half)	28-33	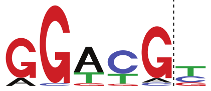
