# Supplementary material for: Conserved DNA sequence features underlie pervasive RNA polymerase pausing
Source: Nucleic Acids Res. 2021 Mar 31;49(8):4402–20. doi: 10.1093/nar/gkab208 (PMC8096220; doi:10.1093/nar/gkab208)
Supplement: gkab208_Supplemental_Files [file gkab208_supplemental_files.zip › SupTable1.pdf]

Supplemental Table 1

**Category**	**Features**
Skewness	AT-, AC-, AG-, CT-, GC-, GT- skewness
Nucleotide identity	At positions +1, -1, -2, -3, -10, -11
Thermodynamics	Entropy, enthalpy, Gibbs free energy, and melting temperature
RNA hairpin	Minimum free energy of nascent RNA fragment
DNA shape	Minor Groove Width, Roll, Propeller Twist, Helix Twist, Potential Energy
DNA structures	Z-DNA, A-phased repeats, inverted repeats, mirror repeats, direct repeats, G-quadruplexes
Transcription factor binding motifs	111 consensus motifs of 639 human transcription factors
RNA binding protein motifs	240 consensus motifs of 160 human RNA binding proteins
Methylation	DNA methylation
