# Supplementary material for: Conserved DNA sequence features underlie pervasive RNA polymerase pausing
Source: Nucleic Acids Res. 2021 Mar 31;49(8):4402–20. doi: 10.1093/nar/gkab208 (PMC8096220; doi:10.1093/nar/gkab208)
Supplement: gkab208_Supplemental_Files [file gkab208_supplemental_files.zip › SupTable2.pdf]

Supplemental Table 2

Species	Genomic region	Number of sites *n*	Number of genomic features *p*	Number of folds *k* used for cross validation	AUC of precision -recall curve	Number of trees	Min samples to split an internal node	Min samples required for a leaf	Number of features considered per split	Max depth of trees	Bootstrap samples for building trees
H. sapiens	Promoter-proximal	7290	670	10	0.85	2015	10	7	Square root of *p*	21	FALSE
H. sapiens	Gene-body	26968	670	10	0.71	3200	5	7	Square root of *p*	138	FALSE
E. Coli	Gene	162946	39	10	0.86	2015	2	7	Square root of *p*	74	FALSE
S. Cerevisiae	Gene	57862	39	10	0.88	1600	10	7	Square root of *p*	54	FALSE
A. thaliana	Gene	5906	39	5	0.86	6400	5	7	Square root of *p*	21	FALSE
